# Prevalence and factors associated with cognitive impairment in Chinese heart failure patients: A pilot study

**DOI:** 10.3389/fcvm.2022.978432

**Published:** 2022-09-23

**Authors:** Qi Niu, WeiHua Liu, FengLing Wang, YanHong Dong

**Affiliations:** ^1^School of Nursing, Shandong First Medical University and Shandong Academy of Medical Sciences, Taian, China; ^2^Linyi People's Hospital, Linyi, China; ^3^Alice Lee Centre for Nursing Studies, Yong Loo Lin School of Medicine, National University of Singapore, Singapore, Singapore

**Keywords:** heart failure, cognitive impairment, Chinese, nursing, prevalence

## Abstract

**Background:**

The prevalence of Cognitive impairment (CI) is high in patients with heart failure (HF). It leads to poor prognosis, such as self-care, hospital readmission and increased mortality. However, such information among Chinese population is unclear.

**Objective:**

The purpose of this study was to examine the prevalence of CI in Chinese patients with HF, and explore its correlation with biomarkers and clinical factors to better manage HF patients with CI.

**Methods:**

This study is a cross-sectional study of 200 hospitalized HF patients in China. The cognitive function of HF patients was assessed by the Montreal Cognitive Assessment (MoCA) and the Mini-Mental State Examination (MMSE).

**Results:**

The majority are male (62.5%, *n* = 125), have primary school and below level of education (57.5%, *n* = 115), NYHA III and above (62%, *n* = 124). They have an average MoCA score of 15.10 ± 8.18, MMSE score of 19.55 ± 8.23. Age, NYHA class, and atrial fibrillation were independently associated with CI (*p* < 0.05). There was a significant association between CI and the 4th quartile of TNT (*p* = 0.013), and the 3rd and 4th quartile of NT-proBNP (*p* = 0.015*, p* = 0.038).

**Conclusions:**

The prevalence of undiagnosed CI in Chinese HF patients is high (81%). HF patients with high levels of TNT or NT-proBNP or both values may be at risk of developing CI. Therefore, we suggest that HF patients with older age, atrial fibrillation, NYHA class II and III, as well as elevated TNT or NT-proBNP or both values to be followed up with a formal evaluation for CI. Nurses need to provide targeted health education program for cognitively impaired HF population to improve their self-care ability and nursing outcome.

## Introduction

Chronic heart failure (HF) is a syndrome resulting from multiple, long-standing cardiovascular abnormalities, such as coronary artery disease or hypertension (1). HF is increasing in prevalence and is a public health problem in the world. Currently, the overall prevalence of HF is 1.3–6.7% in Asian population (2). The China Hypertension Survey (CHS) of 22,158 participants from 2012 to 2015 reported the prevalence of HF was 1.3% (3). There is increasing evidence reported that HF is associated with cognitive impairment (CI) independently (4). CI is highly prevalent in Asian HF patients (44%) (5), but such information in Chinese population is scant. Additionally, CI is closely associated with poor prognosis, such as self-care, hospital readmission and increased mortality (6, 7). The 2016 European Society of Cardiology guidelines focused on self-management of HF patients with CI (8). To better understand self-management of cognitively impaired HF patients, this study examined the prevalence of CI in Chinese HF population and explored its correlation with biomarkers and clinical factors.

## Materials and methods

### Study design and participants

We performed a cross-sectional study of hospitalized HF patients at Linyi People Hospital, Shandong Province, China. We recruited 200 inpatients aged 18 years old. Patients were deemed ineligible if they had significant language or physical impairment impeding their abilities in cognitive tests (e.g., aphasia, hearing and vision impairment, severe hemiplegia, etc.). Patients who had clinically significant psychiatric disorders (e.g., anxiety, depression) hyperthyroidism, hypothyroidism, substance abuse (e.g., alcoholism, drug abuse) were also excluded. This study was approved by the Shandong First Medical University (Shandong Academy of Medical Sciences) Human Research Ethic Review Committee. All participants provided written informed consents.

### Demographic and clinical factors

As part of a research, demographic and clinical factors collected by the researchers, including age, sex, race, New York Heart Association (NYHA) class, troponin T (TNT), N-terminal pro-B-type natriuretic peptide (NT-proBNP), left ventricular ejection fraction (LVEF, determined by echocardiogram performed within the past 6 months), Heart Failure with Reduced Ejection Fraction (HFrEF), Heart Failure with Preserved Ejection Fraction (HFpEF), medical history of vascular diseases [hypertension, coronary artery disease (CAD), diabetes mellitus (DM), stroke, atrial fibrillation (AF), Chronic kidney diseases (CKD)], other risk factors such as smoking and alcohol consumption.

### Cognitive function

Cognitive function was assessed with the Montreal Cognitive Assessment (MoCA) and the Mini-Mental State Examination (MMSE). To correct for education effects, 1 point was added for participants with 12 years of education or less on their total MoCA score (9). Scores on both cognitive tools range from 0 to 30 points, with a lower score reflecting greater CI. A recent study in the Asian HF population suggests a cut-off of < 25 on the MoCA or < 28 on the MMSE for CI (4). Hence, in this study, CI was defined by either MoCA < 25 or MMSE < 28, or both MoCA < 25 and MMSE < 28. The MMSE and MoCA were administered by a trained research personnel.

### Statistical analysis

SPSS22.0 software was used for statistical analysis. To examine the population characteristics of the study, we calculated proportions for categorical variables, means and standard deviations for continuous variables. We compared the characteristics of patients with and without CI, using the independent *t*-tests for continuous variables and the chi-square tests for categorical variables. After adjusted for age, gender, NYHA class, AF, prior stroke, and DM, binary logistic regression was used to analyzed the correlation between CI and other variables. Pearson correlation analysis was used to examine the correlation between MoCA, MMSE and other variables. For NT-proBNP and TNT, quartile measures were used to form 4 groups. The lowest quartile was used as a reference group. Logistic regression analysis was used to analyze the association between CI and NT-proBNP, TNT, other variables.

## Results

### Demographic description

Participants had an average age of 76.11 ± 13.38 years. The majority were male (62.5%, *n* = 125), primary school and below (57.5%, *n* = 115), NYHA III and above (62%, *n* = 124). Cardiovascular risk factors included largely CAD (55%, *n* = 100), followed by smoking (40.5%, *n* = 81), hypertension (38.5%, *n* = 77), AF (33.5%, *n* = 67), alcohol consumption (31%, *n* = 62), DM (29.5%, *n* = 59), prior stroke (15%, *n* = 30), and CKD (12.5%, *n* = 25). These participants had an average of 2.55 ± 1.21 total cardiovascular risk factors. Our HF population had an average LVEF of 42.36 ± 11.09, TNT of 0.09 ± 0.20, NT-proBNP of 4083.57 ± 5663.43. Patients had an average MoCA score of 15.10 ± 8.18, MMSE score of 19.55 ± 8.23. As shown in Table 1, compared with patients without CI, those with CI were older (71.14 ± 10.12 vs. 49.95 ± 11.95 years, *p* ≤ 0.001), had higher proportion of female (42.7 vs. 10.5%, *p* ≤ 0.001), less educated (≤ 12 years of education: 29.6 vs. 97.4%, *p* ≤ 0.001). They also had approximately twice higher rates of AF (37.7 vs. 15.8%, *p* < 0.05) and levels of NT-proBNP (4518.93 ± 6107.31 vs. 2227.54 ± 2416.37, *p* ≤ 0.001).

**Table 1:** Study sample characteristics.

**Variables**	**Whole sample** **(*N =* 200)**	**No CI** **(*N =* 38)**	**CI** **(*N =* 162)**	***p*-value**
Age (years)	76.11 ± 13.38	49.95 ± 11.95	71.14 ± 10.12	**≤0.001**
**Sex**				
Male	125(62.5%)	34(89.5%)	114(70.4%)	**≤0.001**
Female	75(37.5%)	4(10.5%)	48(29.6%)	
**Education**				
Primary school and below	115(57.5%)	1(2.6%)	39(24.1%)	**≤0.001**
Secondary school and above	85(42.5%)	37(97.4%)	26(16.0%)	
**NYHA class**				
NYHA I	12(6%)	3(7.9%)	9(5.6%)	
NYHA II	64(32%)	13(34.2%)	51(31.5%)	0.719
NYHA III	64(32%)	15(39.5%)	49(30.2%)	0.907
NYHA IV	60(30%)	7(18.4%)	53(32.7%)	0.254
**Cardiovascular risk factors**				
Hypertension	77(38.5%)	14(36.8%)	63(38.9%)	0.745
CAD	110(55%)	17(44.7%)	93(57.4%)	0.505
DM	59(29.5%)	10(26.3%)	49(30.2%)	0.802
Prior stroke	30(15%)	6(15.8%)	24(14.8%)	0.757
CKD	25(12.5%)	4(10.5%)	21(13.0%)	0.404
AF	67(33.5%)	6(15.8%)	61(37.7%)	**< 0.05**
Smoking	81(40.5%)	21(55.3%)	60(37.0%)	0.200
Alcohol consumption	62(31%)	17(44.7%)	45(27.8%)	0.126
Total cardiovascular risk factors	2.55 ± 1.21	2.50 ± 1.18	2.56 ± 1.22	0.778
LVEF(%)	42.36 ± 11.09	42.26 ± 9.69	42.41 ± 11.39	0.298
HFrEF (EF ≤ 40%)	104 (52%)	19 (9.5%)	85 (42.5%)	0.787
HFpEF (EF≥50%)	59 (29.5%)	9 (4.5%)	50 (25%)	0.373
TNT	0.09 ± 0.20	0.19 ± 0.39	0.07 ± 0.11	0.479
NT-proBNP	4083.57 ± 5663.43	2227.54 ± 2416.37	4518.93 ± 6107.31	**≤0.001**
MMSE	19.55 ± 8.23	28.50 ± 0.65	17.45 ± 7.77	**≤0.001**
MoCA	15.10 ± 8.18	26.63 ± 1.05	12.39 ± 6.60	**≤0.001**

Values are expressed as mean ± standard deviation, or n (%).

CI, cognitive impairment; NYHA, New York Heart Association; CAD, coronary artery disease; DM, diabetes mellitus; CKD, chronic kidney disease; AF, atrial fibrillation; LVEF, left ventricular ejection fraction; TnT, troponin T; NT-proBNP, N-terminal pro-brain natriuretic peptide; MMSE, Mini-Mental State Examination; MoCA, Montreal Cognitive Assessment. The bold values indicate *p* < 0.05.

### Prevalence of cognitive impairment

The prevalence of CI was 81% based on MoCA < 25, or MMSE < 28, or a combination of both in our HF patients. 80% of patients had MoCA scores < 25, 74.5% had MMSE scores < 28, 6.5% of patients had normal MMSE score (i.e., ≥28) but failed MoCA test (i.e., < 25), 1% of patients who had normal MoCA score (i.e., ≥25) but failed MMSE test (i.e., < 28).

### Factors association with cognition

Multiple regressions showed that age, educational level, NYHA class II and III, history of stroke, CKD, AF and LVEF were independently associated with MMSE and MoCA scores (*p* < 0.05). Logistic regression showed that age, education level, NYHA class, AF and alcohol consumption were independently associated with CI (*p* < 0.05), as shown in Table 2. Regarding cardiac biomarkers, the association between CI and TNT was not significant, while the association between CI and 3rd and 4th quartile of NT-proBNP was significant (*p* = 0.027, *p* = 0.017), as shown in Table 3. Controlling covariates, i.e., age, gender, NYHA class II and III, AF, prior stroke and DM, there was significant association between the 4th quartile of TNT and CI (OR = 0.06, *p* = 0.013), and TNT was negatively correlated with CI. Similarly, there was a statistically significant association between CI and the 3rd and 4th quartile of NT-proBNP after adjustment (*p* = 0.015*, p* = 0.038).

**Table 2:** Association between MMSE, MoCA and CI.

**Variables**	**Correlation with MMSE**		**Correlation with MoCA**		**CI**
	**β**	***p*-value**	**β**	***p*-value**	**OR(95%CI)**	***p*-value**
Age(years)	−0.194	**< 0.001**	−0.193	**< 0.001**	**1.151**(1.054-1.258)	**< 0.05**
**Sex**						
Male						
Female	−0.511	**< 0.001**	−0.147	**< 0.001**	2.065(0.134-31.79)3)	0.603
**Education**						
Primary school and below						
Secondary school and above	0.675	**< 0.001**	0.758	**< 0.001**	0.011(0.002–0.085)	**< 0.001**
**NYHA class**						
NYHA I						
NYHA II	4.225	**< 0.05**	4.035	**< 0.05**	0.026(0.001–0.940)	**< 0.05**
NYHA III	4.090	**< 0.05**	4.928	**< 0.05**	0.012(0.000–0.974)	**< 0.05**
NYHA IV	5.436	**< 0.05**	5.622	**≤0.001**	0.170(0.002–11.589)	0.411
**Risk factors**						
Hypertension	0.096	0.907	−0.168	0.808	1.257(0.229–6.908)	0.792
CAD	1.222	0.177	1.086	0.157	0.478(0.057–4.037)	0.498
DM	0.577	0.535	0.160	0.839	0.538(0.071–4.078)	0.548
Prior stroke	−3.170	**< 0.05**	−2.447	**< 0.05**	0.325(0.033–3.203)	0.335
CKD	3.415	**< 0.05**	2.986	**< 0.05**	0.095(0.003–2.824)	0.174
AF	−2.703	**≤0.001**	−1.435	**< 0.05**	**13.582**(1.249–147.663)	**< 0.05**
Smoking	−0.260	0.827	−0.813	0.419	3.526(0.393–31.670)	0.261
Alcohol consumption	−0.147	0.900	0.786	0.427	**0.070**(0.008–0.604)	**< 0.05**
Total cardiovascular risk factors	−0.217	0.797	−0.217	0.521	1.043(0.778–1.399)	0.777
LVEF(%)	0.113	**< 0.05**	0.083	**< 0.05**	1.006(0.910–1.111)	0.909
HFrEF (EF ≤ 40%)	−0.123	0.082	−0.102	0.152	2.810(0.838–9.422)	0.094
HFpEF (EF≥50%)	0.027	0.705	0.003	0.967	3.428(0.890–13.200)	0.073

CI, cognitive impairment; NYHA, New York Heart Association; CAD, coronary artery disease; DM, diabetes mellitus; CKD, chronic kidney disease; AF, atrial fibrillation; LVEF, left ventricular ejection fraction; TnT, troponin T; NT-proBNP, N-terminal pro-brain natriuretic peptide; MMSE, Mini-Mental State Examination; MoCA, Montreal Cognitive Assessment. The bold values indicate *p* < 0.05.

**Table 3:** Association between cardiac markers (quartiles) and CI.

	**CI (*N =* 38)**	**No CI** **(*N =* 162)**	***p-*value**	**CI (before adjustment)**		CI (after adjustment*)	
				**OR (95%CI)**	***p*-value**	**OR (95%CI)**	***p*-value**
**NT-proBNP**							
1st quartile	590.949 ± 255.610	506.760 ± 217.010	0.243	1	0.057	1	0.062
2nd quartile	1674.400 ± 441.432	1464.091 ± 393.457	0.229	1.672(0.650-4.299)	0.286	2.387(0.454-12.563)	0.304
3rd quartile	3508.558 ± 770.628	2962.000 ± 303.457	**0.01**	3.559(1.154-10.978)	**0.027**	24.211(1.846-317.480)	**0.015**
4th quartile	11216.818 ± 8475.586	7194.667 ± 1501.370	**0.007**	4.159(1.284-13.467)	**0.017**	13.175(1.158-149.887)	**0.038**
**TNT**							
1st quartile	0.017 ± 0.006	0.016 ± 0.006	0.724	1	0.293	1	0.105
2nd quartile	0.031 ± 0.005	0.032 ± 0.004	0.655	0.643(0.206-2.006)	0.447	0.343(0.050-2.336)	0.274
3rd quartile	0.049 ± 0.007	0.045 ± 0.005	0.051	0.451(0.159-1.277)	0.134	0.438(0.071-2.713)	0.375
4th quartile	0.191 ± 0.160	0.620 ± 0.571	**0.042**	0.356(0.110-1.148)	0.084	0.060(0.006–0.557)	**0.013**

CI, cognitive impairment; TnT, troponin T; NT-proBNP, N-terminal pro-brain natriuretic peptide.

For NT-proBNP and TNT, quartile measures were used to form 4 groups.

*Adjusted for age, gender, NYHA class, AF, prior stroke, and DM. The bold values indicate *p* < 0.05.

### Cognitive subtests performance

As shown in Table 4. Compared with patients without CI, a higher proportion of patients with CI did not achieve full scores in 4 domain subtests, i.e., naming (80.0 vs. 2.5%), attention (81.9 vs. 0%), abstraction (81.3 vs. 5.0%) and orientation (74.4 vs. 2.5%) on the MoCA. By comparison, they did not achieve full scores in 2 domain subtests, i.e., orientation (84.6 vs. 2.5%), attention and calculation (74.4 vs. 2.5%) on the MMSE. There were more patients passed MMSE yet failed MoCA than those failed MMSE yet passes MoCA (13 vs. 2, shown in Table 5). We further compared average scores of the domain subtests in these 2 groups. MMSE had no domian subtests that could differentiate these 2 groups, while MoCA had 2 domain subtests (Visuospatital and executive function, Abstraction) differentiated these 2 groups. Overall, MoCA had more domain subtests than the MMSE in differentiating those with and without CI. Additionally, there were more patients passed MMSE yet failed MoCA than those passed MoCA yet failed MMSE.

**Table 4:** Comparisons of Proportions of MoCA and MMSE domain subtest below full scores.

	**CI (%)**	**No CI (%)**	***p***
**MMSE domain subtests** ^**a**^			
Orientation (10) *	84.60	3.00	< 0.001
Registration (3)	30.60	0.00	< 0.001
Attention and calculation (5) *	74.40	3.00	< 0.001
Recall (3)	99.00	73.00	< 0.001
Language and praxis (9)	98.70	60.00	< 0.001
**MoCA domain subtests** ^**b**^			
Visuospatial and executive function (5)	84.60	60.00	< 0.001
Naming (3) *	80.00	2.50	< 0.001
Attention (6) *	81.90	0.00	< 0.001
Language (3)	98.80	85.00	< 0.001
Abstraction (2) *	81.30	5.00	< 0.001
Delayed recall (5)	96.90	95.00	< 0.001
Orientation (6) *	74.40	2.50	< 0.001

^a^MMSE domain subtest full score/details: Orientation (10)—Orientation to place and time; Registration (3)—Repeat 3 words; Attention and Calculation (5)—Serial 7 s; Recall (3)—Recall a list of 3 words; Language and praxis (9)—Sentence repetition, comprehension, reading, writing, copy intersecting pentagons.

^b^MoCA domain subtest full score/details: Visuospatial and executive function (5)—Trail B test, Cube copy, Clock drawing; Naming (3)—Confrontation naming (lion, rhinoceros, camel). Attention (6)—Digit span, Vigilance (tapping at the number 1 in a list of numbers), Serials 7 s; Language (3)—Sentence repetition, verbal fluency; Abstraction (2)—Similarities between 2 items; Delayed recall (5)—Recall a list of 5 words; Orientation (6)—Date, month, year, day, place, city.

*The proportion of subtests below full scores of CI differs from the proportion of subtests below full scores of no CI by more than 50%.

**Table 5:** Comparisons of the average MoCA and MMSE domain subtest scores.

**‘**	**MMSE≥28 &MoCA < 25 (*n =* 13)**	**MMSE < 28** **&MoCA≥25** **(*n =* 2)**	***p***
**MMSE domain subtest** ^**a**^			
Orientation (10)	9.92 ± 0.28	9.50 ± 0.71	0.116
Registration (3)	3.00 ± 0.00	3.00 ± 0.00	**
Attention and calculation (5)	4.92 ± 0.28	4.50 ± 0.71	0.116
Recall (3)	2.15 ± 0.69	1.50 ± 0.71	0.234
Language and praxis (9)	8.15 ± 0.38	8.50 ± 0.71	0.287
**MoCA domain subtest** ^**b**^			
Visuospatial and executive function (5) *	1.77 ± 1.59	4.50 ± 0.71	**< 0.05**
Naming (3)	1.92 ± 0.95	3.00 ± 0.00	0.146
Attention (6)	5.61 ± 0.77	6.00 ± 0.00	0.505
Language (3)	1.77 ± 0.73	2.00 ± 0.00	0.670
Abstraction (2) *	1.61 ± 0.51	2.00 ± 0.00	**< 0.05**
Delayed recall (5)	1.38 ± 0.96	2.50 ± 0.71	0.144
Orientation (6)	5.92 ± 0.28	5.50 ± 0.71	0.116

^a^MMSE domain subtest full score/details: Orientation (10)—Orientation to place and time; Registration (3)—Repeat 3 words; Attention and Calculation (5)—Serial 7 s; Recall (3)—Recall a list of 3 words; Language and praxis (9)—Sentence repetition, comprehension, reading, writing, copy intersecting pentagons.

^b^MoCA domain subtest full score/details: Visuospatial and executive function (5)—Trail B test, Cube copy, Clock drawing; Naming (3)—Confrontation naming (lion, rhinoceros, camel). Attention (6)—Digit span, Vigilance (tapping at the number 1 in a list of numbers), Serials 7 s; Language (3)—Sentence repetition, verbal fluency; Abstraction (2)—Similarities between 2 items; Delayed recall (5)—Recall a list of 5 words; Orientation (6)—Date, month, year, day, place, city.

*Not calculate.

**p < 0.05. The bold values indicate *p* < 0.05.

## Discussion

In this pilot study, the prevalence of undiagnosed CI based on cognitive screening tests in Chinese HF patients was high (81%), which may be due to the following reasons. First, optimal cut-points of MMSE < 28 and MoCA < 25 in our study were based on recently published HF population specific cut-off points (5, 10), rather than previous studies that used cut-off points validated for psychiatric inpatients (MMSE < 24) (11) or those with mild CI (MoCA < 26) (9). Second, age was significantly related to CI, our HF patients were much older than previous studies (e.g., ~20 years older than the sample in Dong et al. (58.7 ± 10.5 years old) (5) and Vellone et al. (66.9 ± 11.7 years old) (12).

TNT and NT-proBNP were early biomarker of cardiac dysfunction (13). Our study has shown that TNT and NT-proBNP were significantly associated with CI, consistent with previous studies by Dong et al. (5), Hilal et al. (14), and Gunstad et al. (15). Pokharel et al. (16) indicated that elevated TNT values were more common in older than in younger patients. Our findings on TNT is consistent with a previous study by Hilal et al. (14). The age of our patients with CI were older and comparable to the sample in the study by Hilal et al. (14) (71.14 ± 10.12 vs. 70.20 ± 9.60 years old). Our sample consisted of HF patients only, different from the study sample from Hilal et al. (14) which consisted of community and memory clinic older adults with few HF patients. Furthermore, our finding shows that TNT value is significantly associated with CI at its fourth quartile (Table 3), although it has a statistically non-significant and lower value in those with CI (Table 1). By comparison, NT-proBNP value is significantly associated with CI at its third and fourth quartile, showing lower threshold. This suggests that NT-proBNP might be a more sensitive cardiac biomarker than TNT in HF population.

The possible mechanisms of NT-proBNP associating with CI are as follows. First, the left ventricular dysfunction and ischemic heart disease not only release these biomarkers (NT-proBNP) but also activates several other inflammatory markers leading to ischemic damage in regions selective to cognitive function (17). Second, high levels of NT-proBNP are associated with endothelial dysfunction, possibly linked to cognitive function (18). Third, the association between NT-proBNP and cognitive dysfunction could be due to subclinical cardiovascular disease (i.e., early atherosclerosis) (19). Fourth, higher level of NT-proBNP is also linked to AF (20) which in turn is associated with reduced cognitive function (17).

Our results indicated that AF was associated independently with CI, which was consistent with the conclusion of Kalantarian et al. (21). Beyond clinically recognized shared risk factors (aging and cardiac function), one of the leading potential mechanisms was the occurrence of silent cerebral infarcts (22). AF was associated with a more than two-fold increase in the risk of developing silent cerebral infarcts (23). Although silent infarcts were not associated with clinically apparent acute neurologic deficits, there was a significant association between silent infarcts and the development of cognitive decline and dementia (24). The other possible mechanism was that AF and CI share a common link with regards to protein misfolding and amyloidgenesis (25). Misfolded atrial natriuretic peptide may lead to the formation and deposition of atrial amyloid fibers in elderly patients with AF. β-Amyloid protein and tau protein forming cerebral plaques which exert cytotoxic effects leading to cerebral atrophy, consequently cognitive decline (26, 27). Studies suggested that the occurrence of Alzheimer's disease was related to hypoperfusion, inflammation, oxidative stress, and endothelial dysfunction, and those factors resulting in an atrial cardiomyopathy which in turn, leads to AF (27).

The cognitive deficits of HF patients were shown in several subtests, especially in domains of orientation, short-term memory, attention, concentration and working memory, language (e.g., naming), and executive functions (e.g., abstraction). The domain subtests in the MoCA seems to be better than the MMSE in differentiating HF patients with and without CI. Cognitive deficits interfere with patients' self-care abilities, such as recognizing worsening symptoms, adhering to complex medication regimens, dietary restrictions and numerous lifestyle modifications. CI may contribute to suboptimal self-care in several ways. Several studies have suggested that deficits in memory, attention, and executive functioning were associated with difficulties adhering to recommendations, because of forgetfulness and poor learning ability (28, 29). Executive functioning affected the ability of HF patients to adapt to treatment and lifestyle regimens, by affecting learning and recall efficiency (30). Decline in language function led to poor understanding of medical instructions and contributes to worse adherence, while poor memory and attention have an adverse impact on daily tasks such as attending appointments, adhering to medication and weighing. Therefore, we should identify HF patients with CI early through routine cognitive screening, so as to develop individualized health education according to their cognitive problems. For example, based on the characteristic deficits on MoCA and MMSE, health education for our HF patients should be simpler and easy to understand, kept at a shorter duration, delivered with frequent repetition to strengthen the recall, etc. If necessary, upon discharge from hospital, closer follow-up and ongoing nursing management should be carried out for HF patients with CI, in order to improve their self-care and prognosis. In the future, we can also carry out health education for HF population with CI through home- or community-based care services and remote monitoring by medical professionals (Wechat, apps and so on), so as to prevent the increase of mortality and readmission rate.

Several limitations require acknowledgment. Firstly, small sample size and single study site limit the generalizability of our findings. Larger and multicenter studies are needed to establish the prevalence of CI in Chinese HF patients. Secondly, this study did not conduct a formal neuropsychological assessment to determine CI due to time constraint. Finally, our study showed that alcohol consumption decreased the incidence of CI in patients with HF, which may be related to cardiovascular benefits of light alcohol consumption (31). However, the amount and types of alcohol consumption were not investigated in our study. Future study should examine the impact of alcohol consumption on CI in patients with HF.

In conclusion, the prevalence of undiagnosed CI in Chinese HF patients is high. HF patients with elevated TNT or NT-proBNP or both values may be at risk of developing CI. Therefore, we suggest that HF patients with older age, AF, NYHA class II and III, as well as elevated TNT or NT-proBNP or both values to be followed up with a formal evaluation for CI. Future study should develop customized health education programs for HF patients with CI, so as to improve their self-care ability and nursing outcome.

## Data availability statement

The original contributions presented in the study are included in the article/supplementary material, further inquiries can be directed to the corresponding authors.

## Ethics statement

The studies involving human participants were reviewed and approved by Shandong First Medical University (Shandong Academy of Medical Sciences) Human Research Ethic Review Committee. The patients/participants provided their written informed consent to participate in this study. Written informed consent was obtained from the individual(s) for the publication of any potentially identifiable images or data included in this article.

## Author contributions

QN designed this study and drafted the manuscript with the help from YD. WL and FW reviewed the manuscript. YD conceptualized this study, contributed to the design and provided critical review of the manuscript. All authors contributed to the article and approved the submitted version.

## Funding

This work was supported by Research program of Humanities and Social Sciences in Colleges and Universities of Department of Education of Shandong province (No. J18RB063) 2018.03-2021.08. YD is the current recipient of a Transition Award from the National Medical Research Council (NMRC/TA/0060/2017), Ministry of Health, Singapore.

## Conflict of interest

The authors declare that the research was conducted in the absence of any commercial or financial relationships that could be construed as a potential conflict of interest.

## Publisher's note

All claims expressed in this article are solely those of the authors and do not necessarily represent those of their affiliated organizations, or those of the publisher, the editors and the reviewers. Any product that may be evaluated in this article, or claim that may be made by its manufacturer, is not guaranteed or endorsed by the publisher.
